# Chemical Modification of Chitosan with Bioactive Molecules: A Sustainable Approach for Advanced Film Development

**DOI:** 10.3390/ijms262110403

**Published:** 2025-10-26

**Authors:** Carolina Muñoz-Núñez, Nuria Gómez-Fernández, Alexandra Muñoz-Bonilla, Marta Fernández-García

**Affiliations:** 1 Instituto de Ciencia y Tecnología de Polímeros (ICTP-CSIC), C/Juan de la Cierva 3, 28006 Madrid, Spain; 2 Facultad de Ciencias Químicas, Universidad Complutense de Madrid, Avenida Complutense s/n, Ciudad Universitaria, 28040 Madrid, Spain; 3 Interdisciplinary Platform for Sustainable Plastics Towards a Circular Economy, Spanish National Research Council (SusPlast-CSIC), 28006 Madrid, Spain

**Keywords:** chitosan, modification, eugenol, antioxidant, films

## Abstract

This study presents the synthesis of a new chitosan (CS) derivative incorporating eugenol (EU), a natural compound known for its strong antioxidant properties, with the aim of comparing its properties to those of the previously described thiazolium-chitosan derivative (CS-MTBAQ), employed as antimicrobial component. The functionalization was achieved through a thiol-ene reaction, enabling the covalent bonding of EU and thiol modified chitosan (CS-SH). After detailed characterization of the resulting derivative (CS-SH-EU), a comparative analysis of its antioxidant activities was conducted, revealing that CS-SH-EU films exhibited 25% higher antioxidant efficiency compared to those with CS modified with MTBAQ. Both derivatives were incorporated into chitosan-based films at 10 wt%, which were further reinforced with chitin nanowhiskers at two concentrations, 1 and 5 wt%. The antioxidant, mechanical and structural properties of these films were extensively evaluated as well as the yellowness index and water vapor transmission. The inclusion of these derivatives containing eugenol and thiazolium groups and the chitin nanowhiskers enhanced the mechanical performance, water barrier properties, and antioxidant activity maintained the visual appearance. The formulation applied as coating on strawberries was able to extend their self-life by creating an effective barrier. The findings evidence that the obtained films present a promising alternative for developing active packaging materials, combining enhanced antioxidant efficiency with excellent mechanical and biodegradable properties.

## 1. Introduction

Chitosan (CS) is a biopolymer obtained from chitin, a natural polysaccharide present in the exoskeletons of some arthropods, crustacean shells and fungi. Its structure consists of a linear chain of glucosamine and N-acetylglucosamine units, which are linked by β-(1→4) glycosidic bonds. Chitosan has attracted considerable attention due to its biocompatibility, biodegradability, and non-toxic nature [1]. Its ability to form films makes this polymer widely applicable in areas such as biomedical applications [2], environmental protection [3], and food packaging [4]. Due to its limited solubility at physiological pH [5] and restricted bioactivity [6,7], CS has been the focus of numerous chemical modifications studied in the literature [8,9] to enhance its functionality, improving the water solubility or adding new properties.

One effective strategy to enhance the bioactivity of CS is through N-acylation, using a coupling system based on carbodiimides, specifically 1-ethyl-3-(3-dimethylaminopropyl) carbodiimide (EDC) in combination with N-hydroxysuccinimide (NHS) [10,11]. This system activates the carboxyl groups of the molecules under study, facilitating their reaction with the amino groups of the polymer. This modification not only improves the solubility of CS under neutral conditions but also allows for the incorporation of functional groups that can confer new properties to the polymer.

Chemically modifying CS with bioactive compounds can improve its properties for different applications. Understanding how these modifications enhance the functionality of CS opens up new possibilities for its use as a promising candidate for developing new components for films and coatings [12,13].

The importance of developing sustainable materials for food packaging has been accentuated in response to the environmental challenges caused by conventional plastics. In this context, CS based films offer a biodegradable and eco-friendly alternative, with additional functional properties such as antimicrobial and antioxidant activities, which are critical for extending the shelf-life of perishable products [14]. The use of these films in food packaging has the potential to reduce food waste by preserving product quality and preventing microbial contamination [15]. However, its inherent limitations, such as poor mechanical strength and sensitivity to moisture, need some chemical modifications and the incorporation of nanoscale reinforcing agents to meet the demands of modern packaging applications [16,17].

This study investigates the potential of chemically integrating eugenol, 2-methoxy-4-(2-propenyl)phenol, (EU) into CS. EU, a natural phenolic compound, is mainly extracted from clove oil [18,19], known for its antioxidant and antimicrobial properties [20,21]. Its ability to neutralize free radicals makes this molecule valuable for various applications, including food preservation [22], cosmetics [23], and pharmaceuticals [24]. The antioxidant properties of EU are particularly significant for preventing oxidative damage in biological systems and food products, making it an excellent candidate for creating functional materials with extended shelf life and enhanced bioactivity.

The incorporation of EU into CS to produce a new derivative polymer, and its subsequent use to create films, aims to improve their suitability for food packaging applications by enhancing their antioxidant capacity [25]. The films could not only protect food from oxidative deterioration, but also offer added value as bioactive materials able to maintain product freshness and quality over extended periods [26,27]. This dual functionality aligns with the growing demand for packaging ideas, environmental preservation, and food security.

In this article, this synthesized derivative was used as an antioxidant component in chitosan-based blended films. Additionally, chitin nanowhiskers (ChNw) were explored to reinforce CS composites [28,29]. Derived from the chitin in crustacean exoskeletons, these ChNw not only enhance the mechanical properties of CS, such as its rigidity and strength, making them a valuable additive for reinforcing films based on biopolymers [30,31,32] but also can increase the antioxidant properties [33].

This study also examines the use of a previously developed CS derivative obtained also by N-acylation, containing a permanent cationic charge, thiazolium molecule derived from the vitamin thiamine B1 (quaternized 4-(2-(4-methylthiazol-5-yl) ethoxy)-4-oxobutanoic acid, MTBAQ) [34], which has demonstrated significant antimicrobial properties as bioactive component. The antioxidant properties of both CS derivatives were evaluated and compared [35,36]. Additionally, CS films incorporating both modified compounds and ChNws were developed, and their mechanical and antioxidant properties were analyzed. The combination of antioxidant and mechanical enhancements aims to provide a precise understanding of how various modifications and additives influence the properties of these films, offering an alternative to conventional packaging materials supporting environmental sustainability.

## 2. Results and Discussion

Previously, CS-MTBAQ was synthesized by chemical modification of CS with MTBAQ using also the EDC/NHS coupling system [34]. In this case, the degree of incorporation was determined by different spectroscopic methods, and the value was 68 ± 1%. ChNws were obtained by acidic hydrolysis of chitin [37].

In this study, CS was modified with EU to enhance its antioxidant properties and its potential application in the packaging industry. Initially, the thiolation of CS was performed with the thioglycolic acid compound, resulting in 0.45 ± 0.01 mmol of thiol moieties immobilized per gram of polymer (CS-SH). Subsequently, a thiol-ene reaction was carried out to modify CS-SH with EU, obtaining the CS-SH-EU derivative, which will be further characterized. The estimation of free thiol groups was conducted in this CS-SH-EU derivative, and no absorbance value was detected, as the solution did not change to a yellow color. This suggests that no free thiol groups were available to react with the DTNB reagent, and therefore, all the groups reacted with the eugenol molecules. Furthermore, the zeta potential of the synthesized CS derivatives was also analyzed. Previously, ZP of CS-MTBAQ was evaluated at physiological pH due to its solubility in water [34]. However, for CS-SH-EU, the measurement required an adjustment to the pH of 4 to ensure the compound remained solubilized. At this pH, the phenolic groups and the low amount of unmodified amine groups are protonated. Under these conditions, CS-SH-EU exhibited a ZP of 43 ± 5 mV, while CS-MTBAQ displayed a significantly higher value of 96 ± 4 mV. This variance highlights the influence of the chemical composition on the surface charge of modified chitosans.

### 2.1. Characterization of CS Modified with Eugenol

#### 2.1.1. Nuclear Magnetic Resonance Spectroscopy

**Figure 1:**
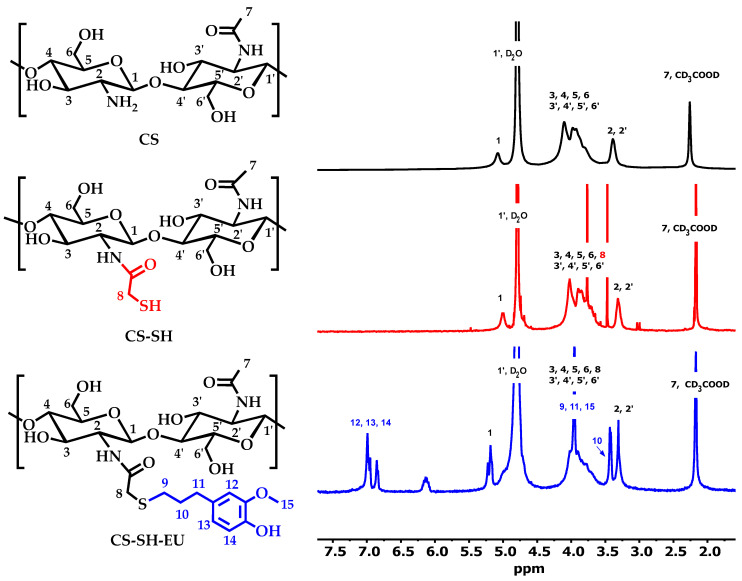
Formula representation and ^1^H-NMR of CS, CS-SH and CS-SH-EU.

Proton NMR analysis was conducted on CS, the compound with the thiol group, and the final product modified with eugenol. This technique provides detailed structural information, allowing for the identification of chemical shifts and interactions specific to each modification. The analysis reveals the molecular changes that occur in each step of functionalization (Figure 1).

First, the ^1^H-NMR spectrum of CS has been widely studied in the literature, and the signals corresponding to protons are well-known. The DA was calculated using the peak areas of H1 proton on the deacetylated ring and the protons (H2–H6) in the N-acetylglucosamine and (H3–H6) in the glucosamine units. The DA obtained was 0.23.

In the case of CS-SH, the DS was obtained using the same relationship, but in this case, the methylene protons of thioglycolic amide were also included in the range between 3.6 ppm and 4.5 ppm. The DS obtained was 0.49, which is an elevated degree of modification [38].

In the spectrum of CS-SH-EU, the aromatic protons are clearly visible around 7.0 ppm. The DM was estimated by the relationship between these aromatic protons and the sum of the protons (H2–H6) in the N-acetylglucosamine, (H3–H6) in the glucosamine units and H7 and H8 methylene protons linked to the amide group. The value obtained was 0.50, which is ca. 65% of deacetylated units. This value confirms the quantitative click reaction.

#### 2.1.2. Fourier-Transform Infrared Spectroscopy

**Figure 2:**
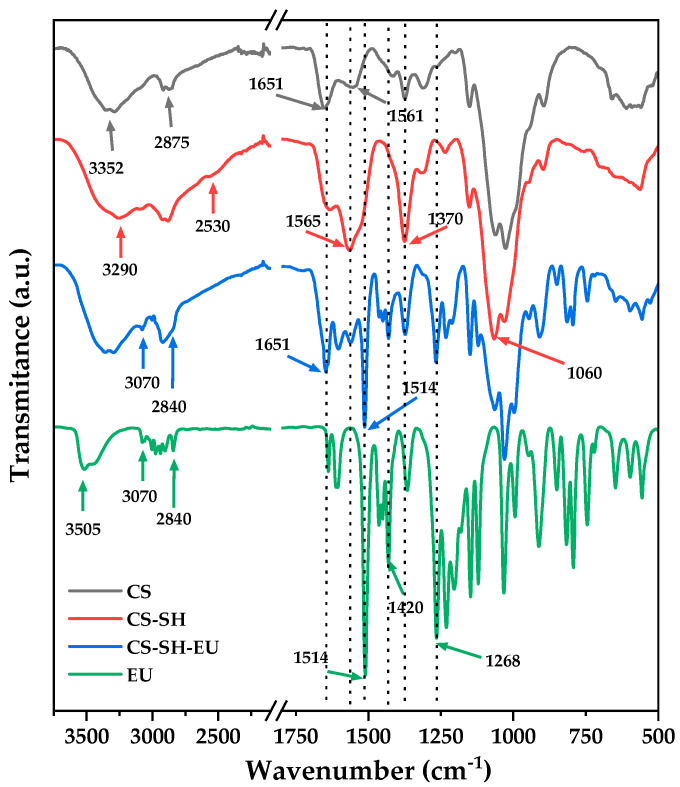
FTIR spectra of CS, CS-SH, CS-SH-EU and EU.

The chemical modification of chitosan was also analyzed using FTIR-ATR, identifying the main functional groups and their interactions. Figure 2 presents the spectra of CS, CS-SH, CS-SH-EU, and EU molecule, where the potential intramolecular interactions can be observed. In the spectrum of CS, a broad absorption band appears at 3352 cm^−1^, corresponding to the stretching vibrations of -NH and -OH groups and the intramolecular hydrogen bonds in the macromolecular chains. The band at 2875 cm^−1^ is attributed to the -C-H stretching vibration. The band at 1651 cm^−1^ is assigned to the carbonyl stretching vibration of the chitosan amide, while the bending vibration of the deacetylated amine is observed at 1561 cm^−1^ [39,40]. In addition, signals between 1300 cm^−1^ and 1050 cm^−1^ are due to the stretching vibration of the -C–O–C- glycosidic bonds [41].

In the case of the first modification of CS with SH, the broad band shifts slightly to 3290 cm^−1^, indicating a more hydrophilic nature. The -CH stretching vibration band remains present, but a subtle new band appears at 2530 cm^−1^, corresponding to the stretching vibration of the -SH group, confirming the formation of the thiol bond [42]. The band at 1565 cm^−1^ becomes more intense, which is attributed to the carbonyl stretching vibration of the introduced SH moieties. Additionally, the band at 1370 cm^−1^ associated with the stretching vibration of the SH group increases [43].

Before discussing the spectrum of CS-SH-EU, it is important to highlight the key bands of the EU molecule. The band at 3505 cm^−1^ corresponds to the -OH group vibration. Around 3070 cm^−1^ and 2840 cm^−1^, the -CH stretching vibrations of sp^2^ and sp^3^ carbons, respectively, can be observed. Another notable band appears at 1514 cm^−1^, corresponding to the -C=C bond in the aromatic ring [36]. The band at 1420 cm^−1^ is attributed to the vibration of the vinyl group interacting with the-CH group.

In the CS-SH-EU spectrum, the characteristic bands of CS and those of the EU molecule are present, confirming that the polymer was successfully modified. It is clear the appearance of bands at 1420 cm^−1^ and 1268 cm^−1^ in the EU incorporation that were not present in CS-SH. Furthermore, the bands associated with the thiol group have either disappeared or significantly reduced (2530 cm^−1^ and 1370 cm^−1^, respectively), providing additional evidence of successful modification.

#### 2.1.3. Elemental Analysis

Elemental analysis of CS, CS-SH, and CS-SH-EU compounds allowed the estimation of DA, DS, and DM. The calculations were based on the percentages of carbon, nitrogen, and sulfur, using Equations (1)–(3), with the results summarized in Table 1. The DA of the crude CS was determined to be 28%, which is close to the 25% acetylation reported by the company, and near to that obtained by NMR. The first modification (DS) was highly successful, but it is higher than that obtained by NMR. Finally, a comparison of DS and DM shows nearly identical values, indicating that the thiol-ene reaction was quantitative as mentioned with NMR data. However, there are some differences between both determinations, which these values have to be taken with attention. Although it is clear the incorporation of EU is around 50% in chitosan.

#### 2.1.4. Thermal Gravimetric Analysis

**Figure 3:**
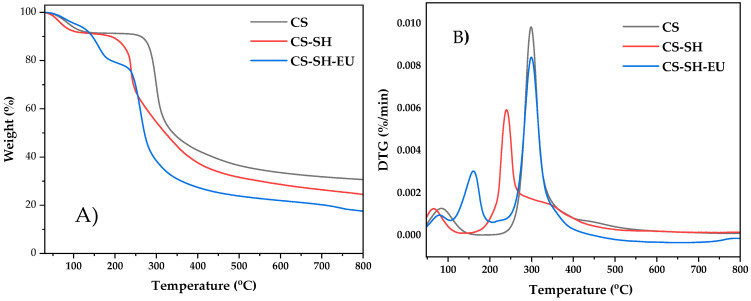
(**A**) Thermogravimetric curves and (**B**) their derivative (DTG) of CS, CS-SH and CS-SH-EU.

The thermal properties of CS, CS-SH, and CS-SH-EU were analyzed using TGA to evaluate the differences in thermal degradation behavior based on the functional groups present in each compound (Figure 3).

For all three samples, a mass loss was observed at temperatures below 100 °C, due to the evaporation of water bound to the chemical structure of CS, primarily through -OH and -NH groups. In the CS sample, a second degradation stage occurred at 257 °C, corresponding to the primary decomposition of glucosamine units up to 392 °C and leading to a total weight loss of approximately 60%. The high carbonaceous residue is attributable to the inert atmosphere used, a common occurrence in carbohydrate polymers [44,45].

In CS-SH polymer, the first step was similar to CS; however, the second thermal degradation began at 190 °C and went up to 271 °C, at lower temperatures than CS, resulting in a 24% weight loss, which corresponds to the decomposition of the thiol groups in the compound [46]. A third stage followed from 271 °C to around 428 °C, corresponding to the degradation of the main CS polymer chains, with around 40% weight loss. Despite this, this polymer produces lower carbonaceous residue than CS.

For the CS-SH-EU polymer, the amount of adsorbed water was lower due to the hydrophobic nature of EU. The second degradation stage started at 120 °C, and continued until 195 °C, leading to a ca. 15% weight loss attributed to the decomposition of the EU attached to CS [47]. Moreover, the third thermal step for this material occurred between 251 °C and 392 °C, relating to the degradation of the main chain, with a total weight loss of 56%. The carbonaceous residue in this polymer is lower than in the former.

#### 2.1.5. Differential Scanning Calorimetry

**Figure 4:**
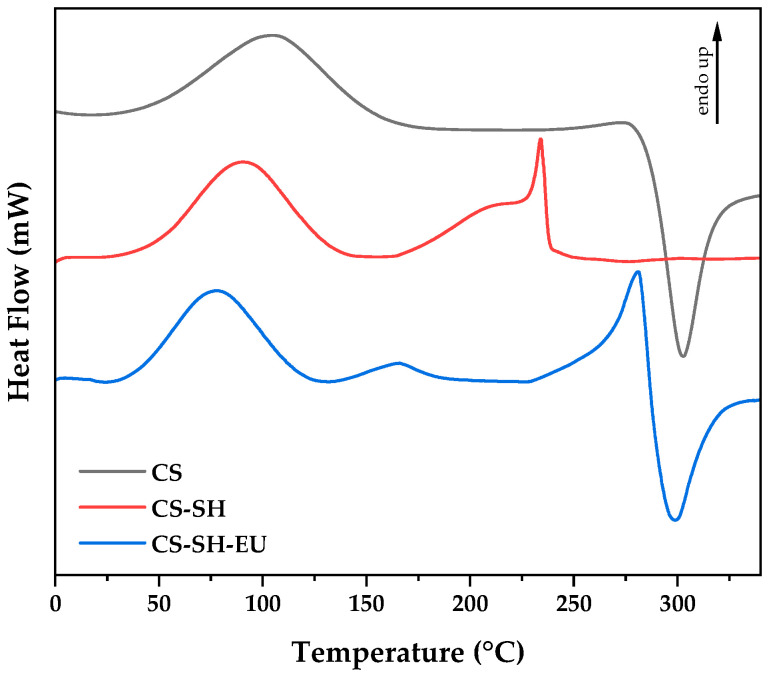
DSC thermograms CS, CS-SH and CS-SH-EU.

DSC studies were conducted to understand the thermal behavior and potential differences between CS and its derivatives, CS-SH and CS-SH-EU (Figure 4). Firstly, the DSC thermogram of CS showed a broad endothermic peak between 20 and 120 °C, corresponding to water loss from the polymer. This feature was observed across all three thermograms studied. Additionally, a second decomposition stage appeared as an exothermic peak between 270 and 350 °C, indicating the degradation of the polymer backbone [48]. Secondly, the thermogram of CS-SH exhibited an endothermic transition at approximately 230 °C, attributed to the decomposition energy of the thiol group linked to the nitrogen of CS. This result reinforces the concept of significant structural changes caused by the chemical modification [49,50].

Finally, in the DSC thermogram of CS-SH-EU, the peak associated with water loss was smaller than in CS and occurred at a lower temperature, suggesting weaker interactions between water and the aryl groups due to eugenol’s hydrophobic nature. A second endothermic peak appeared before the decomposition of the polymer backbone, likely corresponding to the degradation of the eugenol molecules anchored to the thiol group [51,52].

#### 2.1.6. X-Ray Diffraction

**Figure 5:**
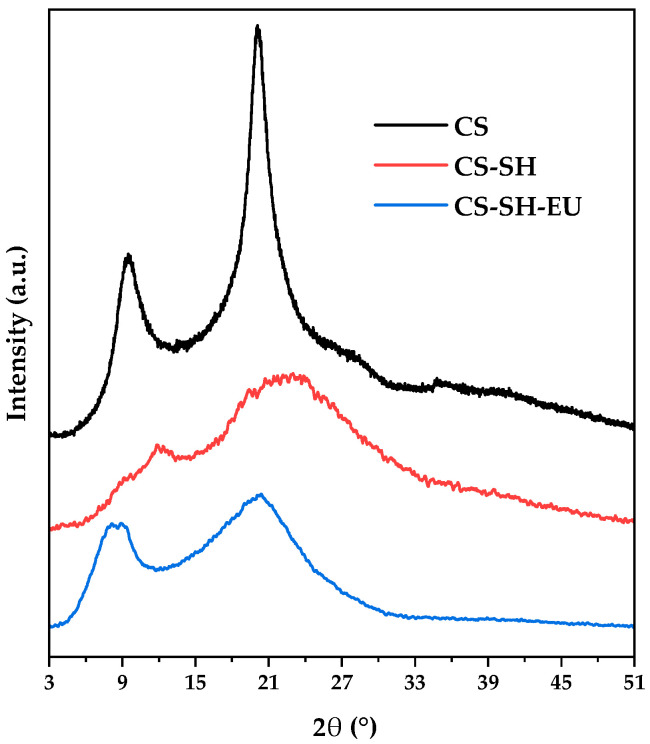
X-ray diffraction patterns of CS, CS-SH, and CS-SH-EU.

The X-ray diffraction patterns of CS, CS-SH, and CS-SH-EU were analyzed to evaluate their crystallinity or amorphous structure, enabling identification of any changes that could affect their mechanical, physical, and chemical properties (Figure 5). The XRD pattern of native CS exhibited a semicrystalline structure, characterized by both ordered (crystalline) and disordered (amorphous) regions. The crystalline regions were associated with the formation of hydrogen bonds between polymer chains, with characteristic peaks observed at 2θ = 9.4° and 20.1°, corresponding to the (020) and (110) crystalline reflections, respectively [53,54]. In the case of CS-SH, it showed significant differences compared to CS, with the main peak shifting from 2θ = 20.1° to 2θ = 23.7° [55]. This shift and broadening of peaks were attributed to the chemical modification of the polymer chain, which introduced new bonds and reduced the crystallinity [56]. Additionally, this derivative displayed a broad peak at 2θ = 13.3°, indicating some residual crystallinity due to the intermolecular and intramolecular bonds formed by the thiol group. In the case of CS-SH-EU, the crystallinity peaks of CS become visible again but with much lower intensity, suggesting that the incorporation of EU changes the principal structure of the polymer [57]. Additionally, a new diffraction peak appears at around 2θ = 8.9°, which could indicate a structural reorganization induced by the introduction of the eugenol molecule. According to the literature [58], this behavior is consistent with the incorporation of some phenolic compounds into the CS, resulting in an ordered arrangement of polymer chains, reflected in the increased crystallinity.

### 2.2. Chitosan Films Containing Functionalized Chitosan Derivatives CS-SH-EU and CS-MTBAQ

Once the CS-SH-EU derivative was prepared and fully characterized, it was employed to modify the properties of CS films. Glycerol plasticizer and ChNw were also incorporated into chitosan films to improve the mechanical properties. In addition, films containing CS-MTBAQ (chitosan modified with thiazolium groups) were also prepared and analyzed for comparison purposes. Then, the resulting films were extensively characterized and evaluated as packaging films and coatings.

#### 2.2.1. Thermal Gravimetric Analysis of Films

**Figure 6:**
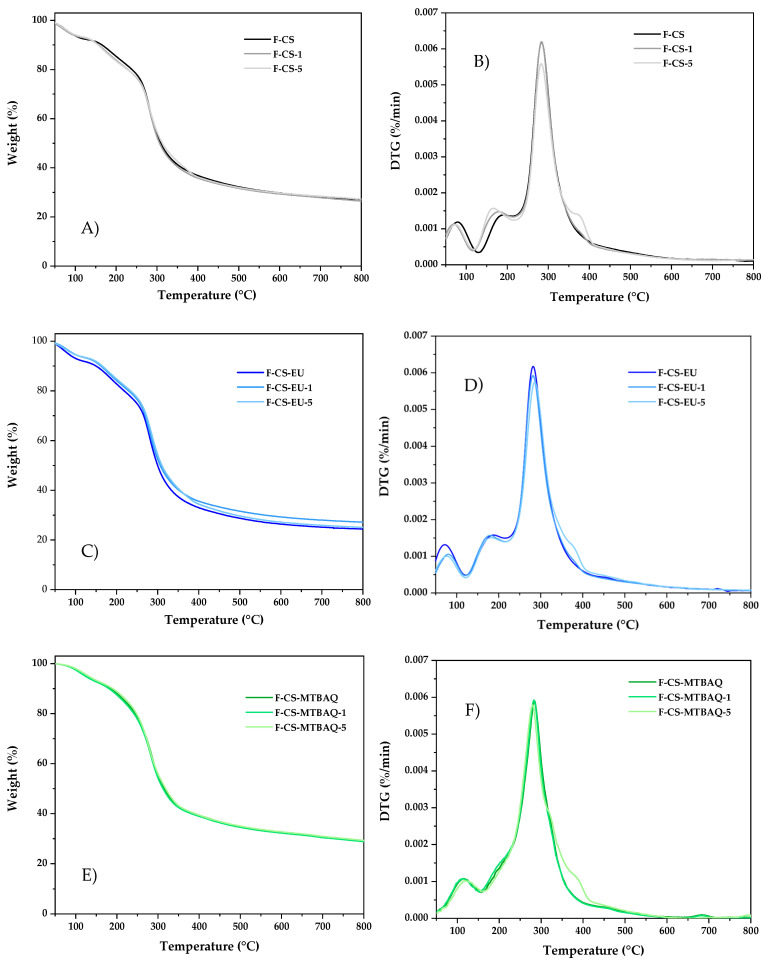
(**A**,**C**,**E**) Thermogravimetric curves and (**B**,**D**,**F**) their corresponding derivative (DTG) curves of the films: F-CS, F-CS-1, F-CS-5, F-CS-EU, F-CS-EU-1, F-CS-EU-5, F-CS-MTBAQ, F-CS-MTBAQ-1 and F-CS-MTBAQ-5.

The thermogravimetric analyses of the films based on CS, CS-SH-EU and CS-MTBAQ, and reinforced with ChNw are presented in Figure 6, along with the corresponding derivative curves. The first stage of thermal degradation corresponded to the loss of residual water in the films. This process began at 37 °C and finished at 125 °C, for F-CS and F-CS-EU films, along with the films with ChNw. However, for the films containing MTBAQ, the weight loss started at 60 °C and continued until 182 °C. This shift was also observed in the second degradation phase. For the CS and CS-SH-EU films, this started at 125 °C and extended to 228 °C, representing the decomposition of glycerol [59,60]. In contrast, in the CS-MTBAQ films, a shoulder appeared around 182 °C, merging with the main degradation peak. This behavior could be attributed to the highly hydrophilic nature of MTBAQ unit, which facilitates interactions between water and glycerol molecules, increasing the energy required for their decomposition [61]. The final stage of thermal degradation, occurring between 228 °C and 350 °C, corresponded to the depolymerization and decomposition of the chitosan units in all samples. Films modified with CS-SH-EU exhibited a greater weight loss during this phase, due to the presence of eugenol molecules. After this stage, films reinforced with 5% ChNw (F-CS-5, F-CS-EU-5, and F-CS-MTBAQ-5) displayed an additional shoulder extending up to 405 °C, attributed to the ChNw incorporated into the films. Similar behavior was observed in CS films with CS modified with methylimidazole [62], but in this case, the second degradation step occurs at lower temperatures than CS films, near 225 °C at the maximum rate.

#### 2.2.2. X-Ray Diffractions of the Films

**Figure 7:**
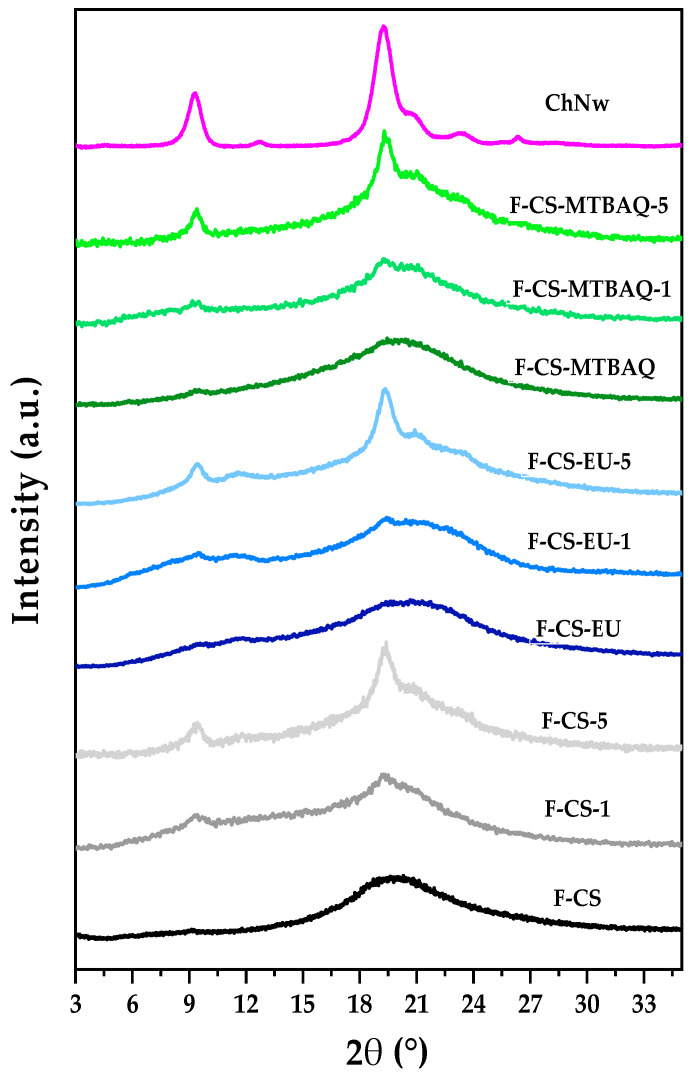
X-ray diffraction patterns of the films: F-CS, F-CS-1, F-CS-5, F-CS-EU, F-CS-EU-1, F-CS-EU-5, F-CS-MTBAQ, F-CS-MTBAQ-1 and F-CS-MTBAQ-5.

The X-ray diffraction patterns for films made from CS, CS-SH-EU, and CS-MTBAQ, along with those reinforced with 1% and 5% of ChNw, are presented in Figure 7. As indicated previously, the profile of CS exhibits a characteristic amorphous peak centered at 2θ = 20.1°. In contrast, ChNws show distinct peaks at 2θ = 8.9° (020 plane) and 19° (110 plane), along with smaller peaks at 2θ = 22° (130 plane) and 26° (013 plane), confirming their consistent crystalline structure [30]. It can be noted that all the films exhibit a broad amorphous peak, pointing to reduced crystallinity caused by the glycerol addition and the inherent film formation. However, with the incorporation of nanowhiskers and an increase in their content from 1% to 5%, the crystallinity increases in all cases. Additionally, there is a rise in the intensity of the diffraction peaks corresponding to the ChNw in all three cases [63,64].

#### 2.2.3. Yellowness Index 

**Figure 8:**
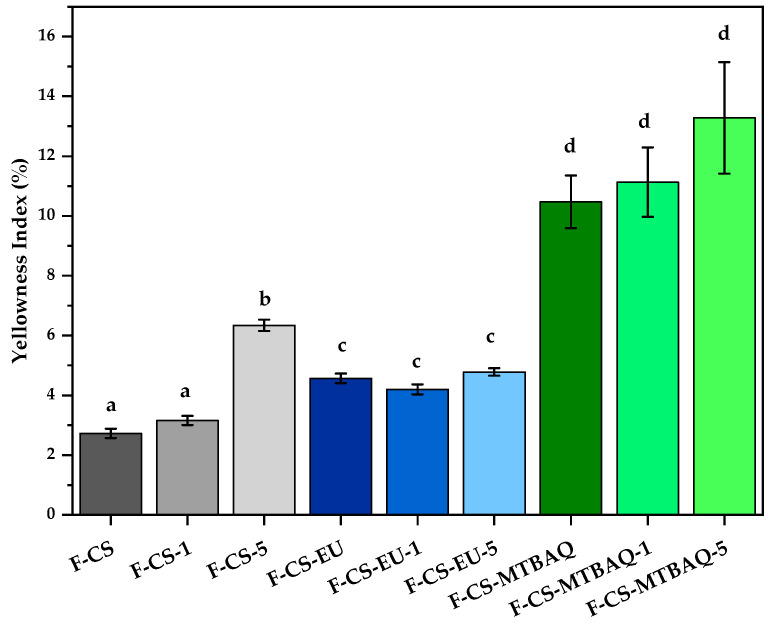
Yellowness index of all chitosan-based films. Letters are used to indicate significant differences among groups (*p* < 0.05).

An important property during the evaluation of the fabricated films for food packaging is their color, and the way that they can influence the consumer acceptance, the freshness and quality of the product. In this study, the YI was used to quantify the visual appearance of the film; the results are shown in Figure 8. Firstly, the films produced only with CS exhibited the lowest YI values. With the incorporation of ChNw, the YI increased with the amount of ChNw added. This effect could be attributed to the higher turbidity of the films, which results in a more yellowish appearance. This phenomenon is common in CS films reinforced with nanoparticles, where this addition affects at scattering and film opacity. In contrast, the films containing CS-EU presented slightly higher values of YI, but no significant increase was observed with the incorporation of ChNw. This could be related to the chemical modification of CS with EU, providing an aromatic group that can stabilize the coloration of the film [65,66]. Some phenolic groups, such as EU, are known to influence the optical properties of polymeric matrices, potentially mitigating the visual impact of the nanowhiskers added. Finally, the films incorporating CS-MTBAQ showed an increase YI, regardless of the addition of ChNw. While the addition of these nanowhiskers increased a little the turbidity the changes were not statistically significant. These results suggest that the yellowness is influenced by the presence of the thiazolium group, and the aromatic structure and properties can contribute to the coloration of the films [67].

#### 2.2.4. Water Vapor Transmission

The barrier properties and water resistance of the films are crucial in evaluating the materials for food packaging and the results are shown in Table 2. Films based on just chitosan (F-CS) showed a slight reduction in WVT with the addition of ChNw. although the differences are not statistically significant. These results were similar to the values reported in the literature [68,69]. In the case of films containing CS-MTBAQ, a considerable increase in WVT values was observed, indicating a deterioration of the barrier capacity. This could be attributed to the greater polarity of the MTBAQ molecule, which increases the affinity for water molecules, facilitating moisture uptake [70,71]. Nevertheless, the incorporation of the nanowhiskers into these films significantly reduced the values of WVT, neutralizing the hydrophilicity caused by the chemical modification. This effect is because of a more compact structure, which limits the mobility of water molecules through the film. This trend suggests an enhancement in the water vapor barrier, due to the increased tortuosity in the diffusion pathway introduced by the ChNw [72]. Conversely, films prepared with CS-EU exhibited the lowest WVT values among all formulations. This reduction can be attributed to the presence of hydrophobic aromatic rings from the eugenol moiety, which decrease the affinity for water vapor [73]. The addition of ChNw also reduced the WVT, suggesting effective dispersion and interaction between the nanowhiskers and the polymer matrix, in the same way of the F-CS-MTBAQ. This interaction likely prevents the formation of voids or channels, improving the film’s compactness and reducing water vapor permeability.

#### 2.2.5. Mechanical Properties

Initially, the mechanical properties of the prepared films were evaluated. The tensile strength of the films produced, as well as the effect of varying ChNw concentrations, were assessed and the results are presented in Table 2. Regarding the Young Modulus, it was higher in films containing modified CS (both CS-SH-EU and CS-MTBAQ). This increase could be explained by the greater flexibility of the derived polymer chains, which allows for different interactions compared to those observed in the CS with deacetylated nitrogen. Additionally and as expected, the Young Modulus increased with the concentration of ChNw, due to new interactions between the nanowhiskers and the film matrix [74]. These interactions improve the material rigidity, resulting in a higher modulus. Secondly, the maximum tensile strength was analyzed. The results indicate that for the films containing CS-MTBAQ, this strength increases, probably due to its structure acting as an anchor that distributes stress more uniformly in the film. However, no significant differences were observed with the addition of ChNw. In contrast, no significant differences were found in the CS and CS-SH-EU films, even with the addition of ChNw, suggesting that it does not substantially impact their strength [75]. Analyzing the elongation at break in the films, distinct trends emerge between CS and its derivatives, CS-SH-EU and CS-MTBAQ. The addition of CS-MTBAQ leads to an increase in the mechanical properties of the forming film. In contrast, the CS-SH-EU derivative shows a significant decrease in the breakage percentage value. This can be attributed to a lower compatibility with the CS matrix due to the hydrophobic nature of CS-SH-EU, creating stress points where the material breaks. The incorporation of ChNw does not significantly modify the ε values in each series.

**Table 2:** Mechanical properties of films: elastic modulus (E), maximal tensile strength (TS), and elongation at break (ε).

Film	E (MPa)	TS (MPa)	ε (%)	WVT 10^−3^ (g/(d × m)
F-CS	11.2 ± 1.6 ^a^	28.7 ± 3.5 ^a^	10.3 ± 3.0 ^a^	81.4 ± 5.4 ^a^
F-CS-1	13.9 ± 2.1 ^a^	24.0 ± 4.9 ^a^	6.4 ± 1.5 ^ab^	78.6 ± 5.5 ^a^
F-CS-5	19.0 ± 2.1 ^b^	24.3 ± 5.7 ^a^	8.9 ± 3.8 ^ab^	70.4 ± 8.7 ^ab^
F-CS-MTBAQ	15.2 ± 3.0 ^ab^	36.9 ± 5.4 ^ab^	15.4 ± 1.9 ^ac^	313.7 ± 32.3 ^c^
F-CS-MTBAQ-1	17.4 ± 2.6 ^ab^	38.8 ± 4.6 ^b^	13.9 ± 3.5 ^c^	103.1 ± 5.6 ^d^
F-CS-MTBAQ-5	16.3 ± 1.6 ^ab^	42.5 ± 4.8 ^b^	17.3 ± 3.0 ^c^	105.7 ± 4.3 ^d^
F-CS-EU	15.8 ± 5.0 ^ab^	30.2 ± 2.7 ^a^	2.8 ± 1.6 ^d^	37.6 ± 0.5 ^e^
F-CS-EU-1	17.6 ± 3.6 ^ab^	29.7 ± 2.6 ^a^	3.3 ± 1.5 ^d^	33.5 ± 1.4 ^f^
F-CS-EU-5	18.2 ± 1.1 ^ab^	25.5 ± 4.8 ^a^	2.1 ± 0.3 ^d^	32.2 ± 0.7 ^f^

Samples with different superscript letters are significantly different at *p* ≤ 0.05.

#### 2.2.6. Antioxidant Properties

**Figure 9:**
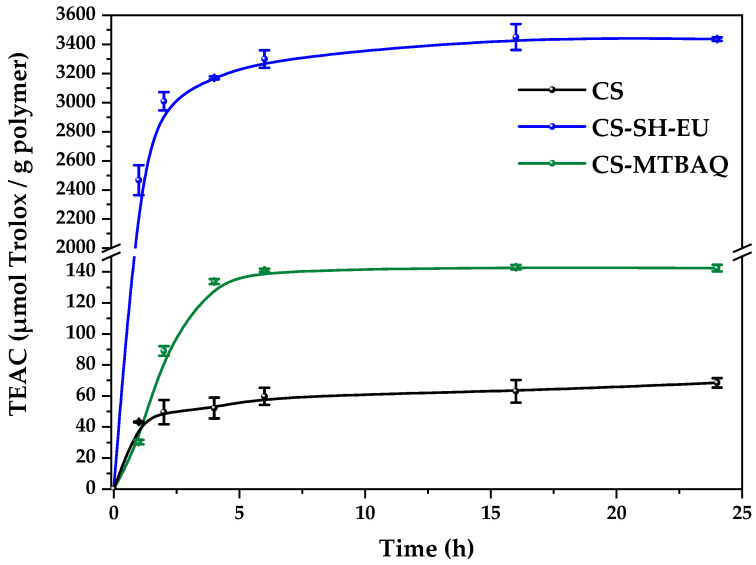
Antioxidant properties of CS, CS-MTBAQ, and CS-SH-EU.

Following the characterization of the CS-SH-EU derivative, its antioxidant capacity was determined, and a comparison was made with the previous CS-MTBAQ system. The study was also performed on the films produced from both systems, which were also reinforced with nanoparticles. Figure 9 shows the antioxidant capacity of these CS derivatives, expressed as Trolox microequivalent antioxidant capacity (TEAC) per gram of sample [76]. 1 mg for both CS and CS-MTBAQ, and 0.04 mg for CS-SH-EU in 1 mL of DPPH solution were used. The results reveal good antioxidant capacity for both derivatives with EU and MTBAQ molecules, reaching their maximum effect 6 h after exposure to the DPPH solution.

The TEAC values were between 3440 and 3450 µmol/g of polymer in the case of CS-SH-EU and around 140–143 µmol/g of CS-MTBAQ. Notably, comparing both results, it can be concluded that the chitosan derivative with eugenol exhibits 25 times greater efficiency than CS-MTBAQ. The antioxidant activities of these two molecules, eugenol and thiazole, differ in effectiveness and mechanism. Eugenol, a phenolic antioxidant, demonstrates high capacity to neutralize free radicals through hydrogen donation, allowing it to fast and effectively inhibit oxidation in biological and food [77]. In contrast, thiazoles, while possessing antioxidant capacity, do so to a lesser extent and via a different mechanism; they act through specific chemical modifications on free radicals, making them less potent in direct neutralization but effective in environments where a more localized and controlled approach is needed [78]. In short, eugenol offers immediate, broad-spectrum antioxidant action, whereas thiazoles exhibit a more specialized and limited capacity in direct comparison.

It is reported that CS could react with free radicals throughout amine groups and generate highly stable ammonium groups and stable macromolecular free radicals [79,80]. In addition, the inter- and intramolecular hydrogen bonds of this polysaccharide reduce free-radical reactions [81,82]. This could be the explanation for the complex behavior of CS and CS with Nw films.

It is also described that gallic acid (GA)-grafted chitosan presents high DPPH scavenging capacity [83,84]. It was compared with free GA, which presents excellent properties at concentrations as low as 0.05 mg/mL. The GA-CS potential is comparable to GA at concentrations of 0.6 mg/mL, which is around 20 times higher than CS. Phenolic derivative chitosans also present good antioxidant properties [85], with the best value reached with 0.9 mg/mL, approximately 10 times higher than CS. Modification with coumaric acid of CS with different molecular weights also produces materials with double radical scavenging capacity than their corresponding pristine CSs [86]. In the present study, CS-SH-EU shows a stronger capacity, with 50 times higher power than the described chitosan polymers.

**Figure 10:**
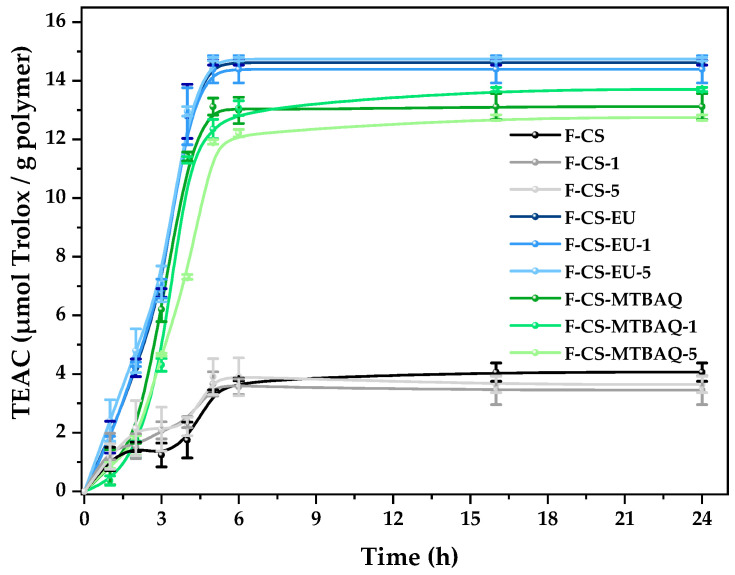
Antioxidant properties of all chitosan-based systems.

Based on this, the antioxidant properties of CS film and its derivatives with 10% of each modified CS (CS-MTBAQ and CS-SH-EU), along with 1 and 5% of nanoparticles, were evaluated. Briefly, 10 mg of each film was weighed and put in contact with 1 mL of DPPH solution; the Trolox equivalents reduced over time were measured through absorbance [26,87]. The results are shown in Figure 10.

For the films containing 10% CS-MTBAQ, antioxidant capacity reached 12–13 µmol of Trolox equivalents per g of film. This was expected, due to the amount of CS-MTBAQ, which makes up 10% of the 10 mg of film (1 mg), resulting in strong antioxidant performance. On the other hand, adding chitin nanocrystals slightly reduced the antioxidant properties, though this reduction was not significant. In the case of the films produced with CS-SH-EU, antioxidant capacity is observed to emerge earlier than in the films produced with CS-MTBAQ. Furthermore, a higher level of antioxidant capacity, measured in Trolox equivalents, is noted compared to the other films. Similarly, the nanocrystals do not exhibit significant capacity, rendering them unremarkable. In these films with chitosan functionalized with eugenol groups, the antioxidant capacity is higher than in the case of incorporation of CS-MTBAQ, due to the stronger ability of CS-SH-EU. The values obtained are in concordance with this assumption.

#### 2.2.7. Coating Application

**Figure 11:**
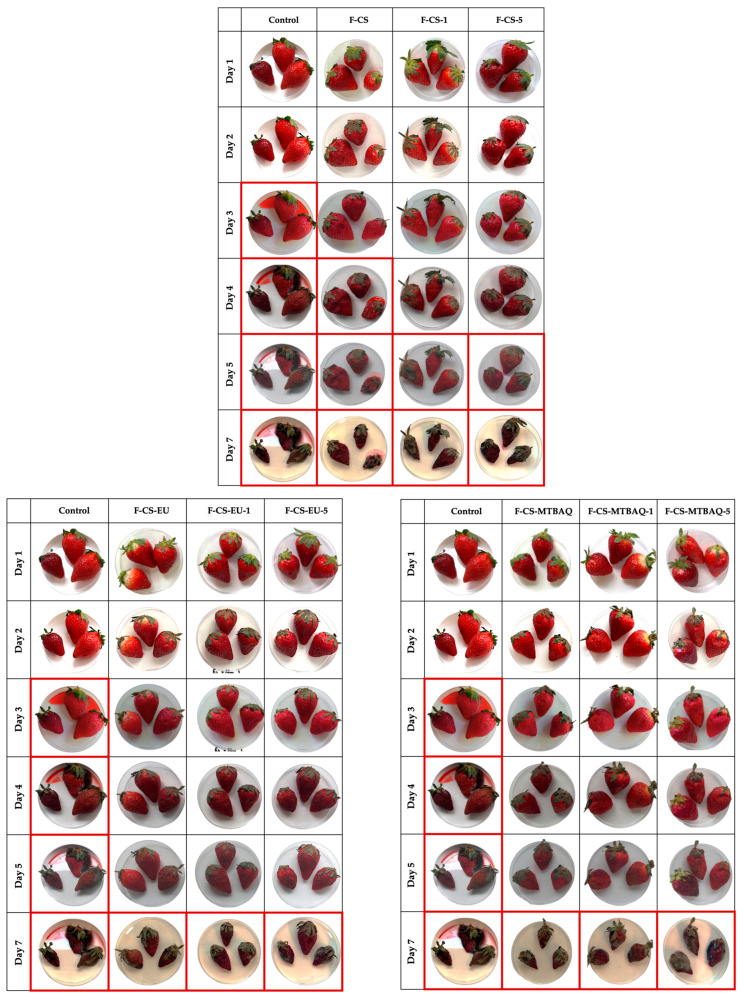
Strawberry evolution over time under various coatings and without treatment.

Based on the mechanical and biological properties evaluated in this work for the F-CS, F-CS-EU and F-CS-MTBAQ, with and without ChNw, these materials could be considered adequate candidates for application as food packaging films or as food preservation coatings. To assess their potential use, strawberries were selected and coated, then the fruits were monitored over time at room temperature to compare the effects of the different coatings against the uncoated control employed as a reference [88,89]. As shown in Figure 11, the absence of a coating in the control group results in a water loss from the third day, which started with a fast-fungal contamination. In contrast, the coated strawberries exhibited delayed deterioration. Samples coated without just chitosan showed initial signs of deterioration on day 4, while strawberries coated with chitosan containing 1% and 5% ChNw remained visibly intact until day 5. The experiment was continued until the fungal growth was observed in all coated samples, which occurred after one week for both CS-EU and CS-MTBAQ-based coatings. These findings suggest that CS-based coatings can delay microbial decomposition, preserving moisture and visual quality more effectively than uncoated samples. Moreover, coatings incorporating CS-EU and CS-MTBAQ (containing eugenol or thiazolium moieties) demonstrated superior preservation performance, supporting their potential as effective biomaterials for food packaging applications.

## 3. Materials and Methods

### 3.1. Materials

CS from shrimp shells (deacetylation degree > 75%), with Mw of 357 ± 10 kDa [62] chitin (Ch, from shrimp shells), succinic anhydride (≥99%), glycerol (Gly, 99%), EDC (≥98%), NHS, (98%), 4-dimethylaminopyridine (DMAP; 99%), EU (99%), 6-hydroxy-2,5,7,8-tetramethylchroman-2-carboxylic acid (Trolox, 97%) 5,5’-dithiobis(2-nitrobenzoic) acid (Ellman’s reagent DTNB; 99%), 2,2-diphenyl-1-picrylhydrazyl (DPPH) and thioglycolic acid (SH, 98%) were purchased from Merck (Darmstadt, Germany). The MTBAQ [34] and ChNw [37] were synthesized according to previous works.

All the organic solvents were of AR grade, glacial acetic acid (AcCOOH), hydrochloric acid (HCl, 37%) dimethyl sulfoxide (DMSO), methanol (MeOH) were provided by Scharlau (Barcelona, Spain). Cellulose dialysis membranes (CelluSepT1) were obtained from Membrane Filtration Products, Inc. (Seguin, TX, USA).

### 3.2. Synthesis of Chitosan Derivative Modified with Eugenol

**Figure 12:**
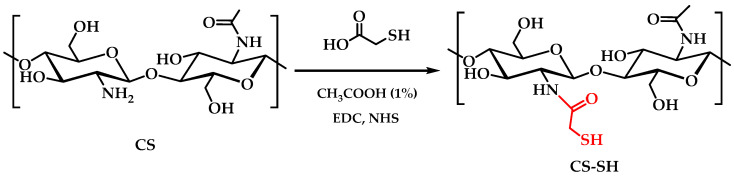
Synthesis of chitosan-thioglycolic acid (CS-SH).

Firstly, chitosan (1 g) was dissolved in 1% acetic acid aqueous solution (*v*/*v*) by constant stirring overnight in a round-bottom flask. The reaction was carried out in the presence of a carbodiimide to enable the amide-forming condensation; for this reason, EDC (1.20 g, 6.26 mmol) was added under stirring. Subsequently, SH (4 mL, 57.16 mmol) was introduced dropwise to the mixture (Figure 12). The pH was adjusted to 4.5–5.0 using NaOH (20 wt%), and the reaction was stirred at room temperature for 24 h [90]. The resulting product was dialyzed against water for removal of unreacted reagents and salts using cellulose membrane tubing (12 kDa) for 4 days at room temperature. Following dialysis, the product was frozen and lyophilized.

**Figure 13:**
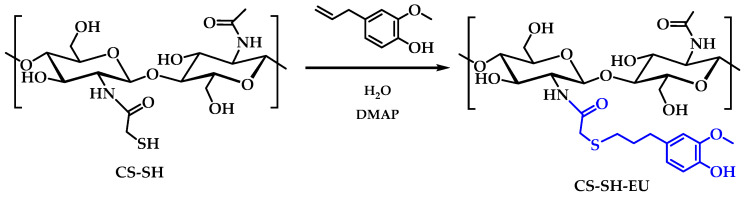
Synthesis of chitosan-eugenol derivative (CS-SH-EU).

CS modified with SH (CS-SH) (0.5 g) is completely dissolved in 20 mL of water. Subsequently, DMAP (0.76 g, 6.26 mmol) is added as a catalyst to mediate the thiol-ene reaction between the thiol group of SH and the double bond of eugenol [91,92]. Once the DMAP is dissolved, EU (1.03 g, 6.26 mmol) was dissolved in 5 mL of DMSO and was gradually added dropwise, with the mixture kept under continuous agitation at room temperature for 24 h (Figure 13). After this period, the reaction mixture turns bright yellow. The resulting solution is then transferred to a tubular cellulose dialysis membrane of 12 kDa and kept under continuous agitation in water at room temperature for four days to remove unreacted EU and DMAP. Finally, the dialyzed product is frozen and lyophilized.

### 3.3. Thiol-Group Determination

DTNB was employed to measure the thiol (-SH) groups chemically attached to chitosan via N-acylation with SH. 3 mg of DTNB were dissolved in 10 mL of 0.5 M phosphate-buffered saline (PBS) at pH 7.5 [93,94]. Then, 0.2 mg of the samples CS-SH and CS-SH-EU were dissolved in 1 mL of the DTNB solution, freshly prepared. The mixture was incubated at room temperature for 60 min to allow the complete reaction between the thiol groups and DTNB. The absorbance of the resulting solution was measured at the wavelength of 412 nm using a BioTek^®^ SYNERGY HTX multi-mode reader spectrophotometer (Winooski, VT, USA). The concentration of thiol groups was determined from a standard curve obtained by cysteine solutions with increasing amounts of thiol groups.

### 3.4. Film Preparation

After synthesizing the CS-SH-EU and CS-MTBAQ products, films were fabricated by casting at room temperature. The final film composition consists of 85% of the polymeric components (CS matrix/functionalized CS-SH-EU or CS-MTBAQ/ChNw) (1 g) and 15% of glycerol (0.18 g). Chitosan was dissolved in a 1% (*v*/*v*) aqueous solution of glacial acetic acid at a solid concentration of 1% (*w*/*v*). CS-SH-EU or CS-MTBAQ were added to the solution at 10% of chitosan’s mass (0.1 g), 1% and 5% quantity of ChNw in relation to CS (*w*/*w*), 0.010 g and 0.050 g, respectively (see Table 3). The mixture was stirred at room temperature for 24 h. Subsequently, 50 mL of each solution was transferred to a Petri dish, and films were obtained by casting. Film thickness was determined at six different points of the film.

**Table 3:** Composition of chitosan films (%).

Films	CS (%)	CS-EU (%)	CS-MTBAQ (%)	ChNw (%)	Gly (%)
F-CS	85	-	-	0	15
F-CS-1	84	-	-	1	15
F-CS-5	80	-	-	5	15
F-CS-EU	75	10	-	0	15
F-CS-EU-1	74	10	-	1	15
F-CS-EU-5	70	10	-	5	15
F-CS-MTBAQ	75	-	10	0	15
F-CS-MTBAQ-1	74	-	10	1	15
F-CS-MTBAQ-5	70	-	10	5	15

### 3.5. Characterization

#### 3.5.1. Nuclear Magnetic Resonance (NMR) Spectroscopy

The proton NMR experiments were carried out at room temperature using D_2_O and CD_3_COOD as solvents on a Bruker Avance III HD-400AVIII spectrometer (Bruker, Rheinstetten, Germany).

#### 3.5.2. Fourier-Transform Infrared Spectroscopy

The infrared spectra of the synthesized materials were acquired on a Perkin Elmer Spectrum Two instrument (PerkinElmer, Waltham, MA, USA) with an ATR sampling accessory. Measurements were taken in the 450 to 4000 cm^−1^ range with a 4 cm^−1^ resolution. A baseline spectrum was obtained before each sample analysis, and all samples were dried under vacuum before testing.

#### 3.5.3. Elemental Analysis

A CHNS-932 PNT01 analyzer (LECO Corporation, St. Joseph, MI, USA) was employed for elemental analysis, allowing for the precise measurement of carbon, nitrogen, sulfur, and hydrogen in the samples. The obtained elemental percentages were utilized to obtain the degree of acetylation (DA) in the case of CS, the degree of substitution (DS) in CS-SH, and the degree of modification (DM) in CS-SH-EU, which were calculated through the following equations [95].

(1) CN=100×n1×Mc+DA×n2×Mc100×n3×MN

(2) CN=100×n1×Mc+DA×n2×Mc×DS×n4×MC100×n3×MN

(3) CN=100×n1×Mc+DA×n2×Mc+DS×n4×MC+DM×n5×MC100×n3×MN

where C/N represents the mass ratio of carbon to nitrogen; M_C_ and M_N_ are the molar masses of carbon and nitrogen, respectively. Whereas, n_1_, n_2_, n_4_ and n_5_ denote the number of carbons of CS deacetylated, acetamido group and SH or EU incorporated into CS with values of n_1_ = 6, n_2_ = 2, n_4_ = 2 and n_5_ = 10. Finally, the n_3_ is the nitrogen number of CS (n_3_ = 1).

#### 3.5.4. Zeta Potential

Measurements of Zeta potential (ZP) for CS derivatives were performed in samples solubilized in distilled water at physiological pH values and room temperature using the Smoluchowski equation to determine electrophoretic mobility. The experiments were performed on a Zetasizer Nano series ZS (Malvern Instruments Ltd., Malvern, UK), and the reported values represent the average of at least ten measurements.

#### 3.5.5. Thermal Gravimetric Analysis

Samples were subjected to thermogravimetric analysis (TGA) using a TGA Q500 thermal analyzer (TA Instruments, New Castle, DE, USA), with a heating rate of 10 °C/min from 25 to 800 °C in an air atmosphere (flow rate of 50 cm^3^/min). Calibration of the instrument was performed according to standard procedures for both temperature and weight measurements.

#### 3.5.6. Differential Scanning Calorimetry

Differential scanning calorimeter (DSC) was conducted on a TA Q2000 instrument from TA Instruments (New Castle, DE, USA), the experiments were performed under a dry nitrogen atmosphere at a flow rate of 50 cm^3^/min. Initially, the samples were equilibrated at 0 °C and subsequently heated to 400 °C at a rate of 10 °C/min. Calibration of temperature relied on the melting points of high-purity chemicals like lauric acid, stearic acid, and indium. Each sample was carefully sealed within an aluminum pan and its mass determined with a high-precision electronic balance, ensuring accuracy to ±0.001 mg.

#### 3.5.7. X-Ray Diffraction

The experimental determination of X-ray (DRX) diffraction patterns for chitosan and its modified compounds was accomplished using a Bruker D8 Advance diffractometer provided with a PSD Vantec detector (Bruker, Madison, WI, USA). CuK_α_ radiation (λ = 0.15418 nm) with an operating voltage of 40 kV at room temperature was used. Samples were securely fixed on a holder and scanned from 2° to 50° (2θ) with a step size of 0.02°, each scan point being collected over a 10 s interval.

#### 3.5.8. Antioxidant Capacity

The antioxidant capacity of CS, CS-SH-EU and CS-MTBAQ was evaluated using the Blois method, which is based on the scavenging of DPPH free radicals [96]. A stock solution of DPPH was prepared by dissolving 6 mg in 250 mL of methanol, stored at 4 °C and protected from light to maintain stability. To assess antioxidant capacity, 1 mL of DPPH solution was added to 1 mg of CS and CS-MTBAQ. However, for CS-SH-EU, 1 mL of the solution was added to 0.04 mg, due to its high antioxidant capacity, to prevent overestimating its capacity. These mixtures were incubated in the dark at room temperature to allow interaction between the DPPH radical and antioxidants in the samples. The absorbance of the solutions was recorded at 517 nm with a BioTek^®^ SYNERGY HTX multi-mode reader spectrophotometer (Agilent Technologies, Winooski, VT, USA) at specific times (1, 2, 4, 6, 16, and 24 h). The DPPH radical’s percentage inhibition was calculated with this equation:

(4) $$I\left(\%\right)=\left(\frac{A_{control}-A_{sample}}{A_{control}}\right)\times 100$$

A_sample_ is the absorbance of the sample after incubation with DPPH, and A_control_ is the absorbance of the control (DPPH solution without antioxidant sample).

Following the evaluation of antioxidant properties in the modified polymers, the capacity of the produced films was assessed using the same method as before. For this analysis, 1 mL of the previously prepared DPPH standard solution was added to 5 mg of each film sample. Absorbance measurements were conducted at 517 nm at specified time intervals to monitor the color change. Radical scavenging activities were standardized and reported in terms of Trolox equivalents. A calibrated curve of Trolox concentration versus inhibition percentage (I%) was used to quantify the antioxidant capacity of the samples, I% = 0.6191 × [C(µM)]. All experiments were conducted in triplicate to ensure the reliability and reproducibility of the data obtained.

#### 3.5.9. Yellowness Index

The chromatic characteristics of the films were determined by measuring transmittance using a PerkinElmer LAMBDA 1050+ UV/Vis/NIR spectrophotometer (PerkinElmer, Waltham, MA, USA). The measurements were taken at a wavelength of 420 nm, as this value is commonly used to assess the yellowness index. Measurements were evaluated under standard illuminant D65 and a 10° standard observer angle. For each sample, three different regions of the film surface were analyzed to ensure representative data. The Yellowness Index (YI) was calculated under ASTM D1925-70 [97], using the following equation:

(5) $$YI=\frac{100\times \left(C_{x}X-C_{z}Z\right)}{Y}$$

CIE color space coordinates (X, Y, Z) and C_x_ (1.3013) and C_z_ (1.1498) are coefficients determined by illuminant. YI provides information of the degree of yellow tonality, often associated with oxidation processes or the presence of others compounds.

#### 3.5.10. Water Vapor Transmission

ASTM E96-00e1 was used to measure the water vapor transmission (WVT) of the films gravimetrically, which allowed for determining their water permeability [98]. Briefly, WVT of the films was measured in permeability cups of an exposed area (A) of 10 cm^2^. To ensure 0% relative humidity (RH) below the film, the cups were filled with 2 g of dried silica gel. These permeability cups were then kept in a desiccator at 23 ± 1 °C and 75% RH maintained with a saturated KNO_3_ solution. For each film formulation, three samples were weighed every hour for the first 8 h, followed by a final weighing at 24 h. WVT (g days^−1^ cm^−2^) was determined using Equation (6),

(6) $$WVT=\frac{slope(g/h)}{A\left(m^{2}\right)\times t(h)}\times thickness\left(m\right)$$

where m_t_ is the mass of the cup at time t, m_0_ is the initial mass of the cup, C is the slope of the mass gain of the dish versus time, and A is the exposed area of the film.

#### 3.5.11. Mechanical Properties

The mechanical properties of the films CS, CS-SH-EU, and CS-MTBAQ were studied to examine the differences when introducing 1 wt% or 5 wt% of ChNw. All films were evaluated through tensile tests at 25 °C with a DX2000 QTest Elite MTS dynamometer (MTS Systems, Eden Prairie, MN, USA), operating at a stretching speed of 10 mm/min. Each specimen was cut into a dog-bone shape, with dimensions of 20 mm in length, 2 mm in width, and roughly 0.1 mm in thickness. A minimum of five specimens per material were tested to ensure reliable results.

### 3.6. Coating Application

Strawberries were provided from a local fruit store and were selected, ensuring a uniform appearance and size, and free of any bruise, to evaluate the preservation effect of the developed coating. Each strawberry was dipped for 1 min in 100 mL of the corresponding solution used for each film formation. Then, the excess of coating material was drained, and the fruits were air-dried at room temperature for 2 h and 25–30% RH [99]. Once dry, they were transferred to separate Petri dishes. Uncoated samples were used as controls, and all experiments were carried out in triplicate.

### 3.7. Statistical Analysis

Experiments were performed in replicates to ensure the reliability of the results. A one-way analysis of variance (ANOVA) was used to identify significant differences, followed by Tukey’s multiple comparison test. Statistical significance was considered at a level of *p* ≤ 0.05.

## 4. Conclusions

The incorporation of the eugenol molecule into chitosan was successfully achieved, providing a chitosan derivative with potential application in food packaging. The CS modification was around 65%, which means that nearly 87% of the deacetylated part was transformed into hydrophobic. Although the introduction of thiol groups destroyed the CS crystallinity, the posterior incorporation of EU provokes a certain order. The antioxidant capacity of this derivative is 25 times higher than that of another derivative containing thiazolium groups, CS-MTBAQ. The inclusion of 10% of both CS-SH-EU and CS-MTBAQ into CS-based films improved the properties of chitosan as food packaging materials. The incorporation of such derivatives produces changes in the mechanical properties of the films, while the incorporation of ChNw does not significantly modify their performance. In addition, the films with CS-MTBAQ, having thiazolium groups, present good antioxidant behavior in comparison with chitosan alone; while CS-SH-EU containing films present quite potent antioxidant character, which does not leach over time, since it is already chemically attached to the chitosan. Other properties, including yellowness and WVT, also support the enhancement of these obtained films, which demonstrated extending the shelf-life of strawberries. These characteristics make these CS-based materials appropriate coating systems for food protection.

## Figures and Tables

**Table 1:** Elemental analysis for carbon, hydrogen, nitrogen, and sulfur. Acetylation degree (DA), substitution degree (DS), and modification degree (DM).

Sample	C (%)	H (%)	N (%)	S (%)	C/N	DA (%)	DS (%)	DM (%)
CS	40.6 ± 0.3	6.7 ± 0.3	7.2 ± 0.3	-	5.6	28	-	-
CS-SH	36.2 ± 0.1	6.4 ± 0.1	5.8 ± 0.1	6.6 ± 0.2	6.2	28	63	-
CS-SH-EU	53.6 ± 0.2	7.1 ± 0.1	5.1 ± 0.1	5.6 ± 0.2	10.5	28	-	62
CS-MTBAQ [34]	33.1 ± 0.3	5.9 ± 0.3	4.8 ± 0.3	5.2 ± 0.3	6.9	28	-	68
ChNw	43.5 ± 0.6	6.4 ± 0.6	6.4 ± 0.6		6.8	-		

## Data Availability

The data availability will be under request.
